# iNOS dependent and independent phases of lymph node expansion in mice with TNF-induced inflammatory-erosive arthritis

**DOI:** 10.1186/s13075-019-2039-z

**Published:** 2019-11-14

**Authors:** Richard D. Bell, Pamelia N. Slattery, Emily K. Wu, Lianping Xing, Christopher T. Ritchlin, Edward M. Schwarz

**Affiliations:** 1 Center for Musculoskeletal Research, University of Rochester Medical Center, 601 Elmwood Avenue, Box 665, Rochester, NY 14642 USA; 2 Department of Pathology and Laboratory Medicine, University of Rochester School of Medicine and Dentistry, Rochester, NY USA; 3 Department of Biology, University of Rochester School of Medicine and Dentistry, Rochester, NY USA; 4 Department of Microbiology & Immunology, University of Rochester School of Medicine and Dentistry, Rochester, NY USA; 5 Division of Allergy, Immunology, Rheumatology, Department of Medicine, University of Rochester School of Medicine and Dentistry, Rochester, NY USA; 6 Department of Orthopaedics, University of Rochester School of Medicine and Dentistry, Rochester, NY USA

**Keywords:** Inducible nitric oxide synthase (iNOS), Inflammatory arthritis, Lymph node, TNF

## Abstract

**Introduction:**

A pivotal effect of lymphatic vessel (LV) function in joint homeostasis was identified in the tumor necrosis factor-transgenic (TNF-Tg) mouse model of rheumatoid arthritis (RA). Specifically, loss of LV contractions is associated with progressive synovitis and erosions. Furthermore, draining lymph node expansion is a biomarker of arthritic progression, and both macrophages and lymphatic endothelial cells express inducible nitric oxide synthase (iNOS), which disrupts LV contraction and transport of immune cells to the draining lymph nodes. Therefore, to directly assess these relationships, we tested the hypothesis that TNF-Tg mice with global genetic ablation of iNOS (iNOS^−/−^) will show delayed draining lymph node expansion, maintained LV contractions, and decreased synovitis and erosions.

**Method:**

iNOS^−/−^× TNF-Tg female and male mice, and control littermates (iNOS^−/−^, TNF-Tg, and WT), were examined with (1) ultrasound to determine popliteal lymph node (PLN) volume and (2) near-infrared imaging (NIR) to assess popliteal LV contraction frequency, and differences between genotypes were assessed at 3, 4, 5, and 6 months of age. Knees and PLN were harvested at 4 months in females and 6 months in males, to assess synovitis, bone erosions, and cellular accumulation in PLN sinuses via histology.

**Results:**

Initially, an increase in PLN volume was observed for both female and male iNOS^−/−^× TNF-Tg and TNF-Tg compared to their WT and iNOS^−/−^ counterparts at 2 and 3 months, respectively. Subsequently, TNF-Tg PLNs continue to increase in volume, while iNOS^−/−^× TNF-Tg did not increase in volume from the initial timepoints. WT and iNOS^−/−^ PLN volume was unchanged throughout the experiment. LV contraction frequency was increased at 4 months in females and 5 months in males, in the iNOS^−/−^× TNF-Tg mice compared to the TNF-Tg. Synovitis and erosions were moderately reduced in iNOS^−/−^× TNF-Tg versus TNF-Tg knees in females, while no differences in knee pathology were observed in males.

**Conclusions:**

Genetic iNOS ablation maintains draining lymph node volume and LV function during TNF-induced inflammatory arthritis and is associated with moderately decreased joint inflammation and damage.

## Introduction

Rheumatoid arthritis (RA) is a chronic, debilitating, inflammatory disease of diarthrodial joints that affects 1.0% of the adult population worldwide [1]. The hallmark characteristics include immune cell infiltration to the synovium accompanied by proliferation of resident fibroblast-like synoviocytes (FLS), leading to hyperplasia of the normally delicate synovial membrane. The resulting “synovitis” forms an advancing destructive tissue that erodes cartilage and resorbs bone [1]. In the clinic, patients often present with pain and swelling of affected joints and, in severe cases, progressive loss of function in affected joints.

During the course of inflammatory arthritis, excess fluid, cells, and macromolecules produced in inflamed joints are cleared from the interstitium by the synovial lymphatic system [2]. Importantly, the lymphangion, the functional unit of the lymphatic collecting vessels, generates tone and intrinsic pulsatile contractions that propel fluid from which progresses towards draining lymph nodes (LNs). These contractions are generated by a tightly controlled cascade of signaling molecules to generate the required tone and subsequent contractions followed by relaxation of the vessel [3]. The balance between Ca^2+^ flux which shortens myosin filaments and nitric oxide (NO) release which lengthens the filaments regulates lymph fluid movement via a series of contractions. Pathologic disruptions of these contractile events may contribute to buildup of fluid during chronic inflammation.

Recent studies in the tumor necrosis factor-transgenic (TNF-Tg) mouse model of RA demonstrated a critical role of the lymphatic system in joint homeostasis [2]. Detailed analysis of joint draining lymph nodes revealed dynamic cellular and volumetric changes that correlated with progression of arthritis [4–8]. These mice show marked abnormalities in both the volumetric changes in the LN over time and in the function of the lymphatic vessels. Imaging of the popliteal lymph node (PLN) identified an expansion phase that began at 2.5 months of age and peaks between 5 and 6 months of age. Serial recordings during this time frame showed a steady lymphatic contraction frequency, with the lymph pumping pressure and CD11b+ cell velocity increased compared to a control mouse [5, 6, 8]. This expansion phase is followed by a temporally stochastic collapsing phase after 5 months of age, characterized by a dramatic reduction of LN volume, with CD23^+^/CD21^hi^ B cells filled sinuses, and a loss of vessel contraction and stagnant CD11b+ cells within the vessel [8, 9]. The serial phases of LN volume changes are accompanied by an augmentation of synovial volume. TNF-Tg mice with expanded LNs have larger synovial volumes than WT mice, and TNF-Tg mice with collapsed LNs have the largest synovial volumes. The collapse of the nodes is also associated with elevation of the power Doppler signal, a measure of increased blood flow [5, 7, 9]. Histology and micro-computed tomography analyses show that severe knee synovitis and bone erosion do not occur until the PLN collapses, indicating PLN collapse is a biomarker for severe knee arthritis [7, 8, 10]. Importantly, all of these studies were performed in male mice, but when we investigated female mice, we found significantly altered progression indicating that in the TNF-Tg mouse model, these lymphatic and arthritis phenotypes are sexually dimorphic [11]. Female mice show an earlier peak expansion (~ 4 months of age) and collapse (~ 6 months of age) of their popliteal lymph node concomitant with earlier starting synovitis and bone erosions (3 months of age). These results suggest the TNF-Tg mouse model is ideal to study the lymphatic disease phenotype in the context of arthritis and that sex differences in the progression of disease should be considered.

Another finding of particular relevance to the potential contribution of the lymphangion in RA is that lymphatic vessels (LVs) undergo significant pathologic changes throughout the course of arthritis, which ultimately leads to the loss of lymphatic function. It is well known that monocytoid cells migrate to and proliferate in the synovium during inflammatory arthritis [12, 13]. Intravital fluorescent imaging demonstrated that during the expansion phase, CD11b+ cells stream away from the joint in LV, and during the collapsed phase, they are largely immobile in the vessel [7, 8, 10]. Monocytes and macrophages are also primary producers of inflammatory mediators, such as TNFα and iNOS, and catabolic factors (e.g., matrix metalloproteases) [1, 13]. Thus, stationary-activated macrophages within LVs adhere to lymphatic endothelial cells (LECs), permeabilize the endothelium, and promote apoptosis of lymphatic muscles cells (LMCs), as evidenced by transmission electron microscopy [7, 14]. Furthermore, MRSA-induced lymphatic muscle cell loss does not recover after 260 days suggesting a prolonged defect if the LMC coverage deteriorates [15]. Evidence for a role of iNOS in LV loss of function in arthritis comes from studies demonstrating inflammation-induced iNOS in LECs and recovery of LV contractions following chemical inhibition of iNOS [16, 17]. iNOS inhibition also improves lymphatic function in obese mice, a chronic inflammatory setting [18]. Taken together, these findings indicate that chronic inflammation induces structural and functional changes to LVs, including the overproduction of iNOS, which inhibits LV contractility and lymphatic drainage from inflamed joints. This marked decline in LN function may be the trigger for volumetric collapse of the draining LN and induce arthritic exacerbation [2]. Therefore, in this study, we examine the premise that global genetic ablation of iNOS will delay or block draining LN expansion, maintain LV contractions, and lessen the severity of synovitis and erosions in a murine RA model.

## Methods

### Animals

The present work was conducted with prior approval of the University of Rochester Medical Center University Committee for Animal Resources. The 3647 tumor necrosis factor-transgenic mouse lines were originally obtained from Dr. George Kollias [19, 20], are maintained as heterozygotes, and have been maintained across multiple generations. iNOS^−/−^ mice were obtained from Jackson Laboratories (strain: B6.129P2-*Nos2*^*tm1Lau*^/J) and were crossed with TNF-Tg mice to obtain iNOS^−/−^× TNF-Tg^+/−^. All studies were performed with littermate wild-type or iNOS^−/−^ controls. Due to the observed temporal differences in male and female TNF-Tg pathology [11], male and female animals were studied at different ages to match disease phenotype. Longitudinal analysis (ultrasound and near-infrared lymphatic imaging) was performed in female mice at 2, 3, and 4 months of age and in male mice at 3, 4, 5, and 6 months. The mice were subsequently euthanized, and tissues harvested for histologic analysis.

### Ultrasound

B-mode and power Doppler ultrasound imaging was performed with the VisualSonics Vevo3100 using the MX700 probe (FUIJIFILM, Toronto, ON, Canada) on the left and right PLN. LN volumes and normalized power Doppler (PD) signal were collected as previously described [6, 7, 14, 21]. Briefly, mice were anesthetized with 1–3% isoflurane, depilatory cream was applied to remove hair from the lower limbs, and the mice were restrained under anesthesia on a thermal bed maintained at 37 °C. The foot pads were affixed to electrodes to measure the heart and respiratory rate. B-mode and power Doppler (PD) images were obtained between breaths to avoid motion artifact. The Doppler parameters were as follows: frequency, 40 MHz; power, 100%; PRF, 1.5 kHz; gate, 6; Doppler gain, 34 dB; gain, 32 dB; depth, 10 mm; width, 5.73 mm; bean angle, 0°; sensitivity, 5; and line density, high. B-mode and PD image stacks were imported to Amira (ThermoFisher Scientific, Hillsboro, OR, USA), and the LN was segmented using the B-mode images to obtain lymph node volumes. PD signal within the LN segment was then quantified and normalized to total LN volume (NPDV).

### Near-infrared lymphatic imaging

Near-infrared imaging was performed as previously described [2, 8–10, 16, 21, 22]. Briefly, mice were anesthetized with 1–3% isoflurane, and depilatory cream was applied to remove hair from the lower limbs and then maintained under anesthesia on a thermal bed at 37 °C. Ten microliters of 100 μg/ml indocyanine green was injected into the left and right foot pads, and feet were restrained at a 45° angle to the body to visualize the popliteal lymphatic vessels. Near-infrared images were captured on a custom-built system 40 min after injection in order for indocyanine green (ICG) to fill the lymphatic vessels and return to homeostatic interstitial pressures following application of tissue pressure to restrain the hind limb as increased interstitial pressure alters lymphatic contractility [23]. Images were recorded over 10 min and imported into Image J for region of interest (ROI) analysis. The ROI was affixed over a lymphatic vessel and boluses of dye travel through it over time to generate ICG intensity graph over time. Lymphatic contraction frequency was calculated by scoring the graphs for peaks and valleys (performed by RDB).

### Histology

At the terminal endpoints (4 months of age in females and 6 months of age in males), popliteal lymph nodes and knees were fixed in neutral-buffered formalin and processed for paraffin-embedded histology. Sagittal sections of the knees and ankles were obtained, and one section from the medial compartment was stained with hematoxylin and eosin (H and E), and for tartrate-resistant acid phosphatase (TRAP), as we have previously described [24]. B220-PE antibody was used to stain PLNs for B cells. All slides were scanned with an Olympus VS120 (full slide scans available upon request), and histomorphometry was performed on the resulting digital images. Total lymph node area, sinus area, and cell area within the sinuses as well as total synovial area and cell area within the synovium were quantified with Visiopharm (Hoersholm, Denmark) using an application adapted from the H and E lung analysis previously described [25].

### Statistics

All statistical analyses were performed in JMP Pro 13 (SAS, Durham, NC). All continuous variables were analyzed for normality via a Shapiro-Wilk test. For outcomes that were not normally distributed, a rank transformation was performed before further analysis. Mixed models for the longitudinal variables weight, PLN volume, NPDV, and contraction frequency with a repeated structure were used to assess the interactions between different combinations of factors (age, TNF-Tg presence, and iNOS^−/−^ presence) when appropriate. When significant three-way interaction effects were observed, Tukey’s post hoc tests were performed to investigate specific differences. When significant interaction effects were not observed, custom contrast *t* tests were performed between TNF-Tg mice with and without a functional iNOS gene at each timepoint with an adjusted alpha level of 0.016 to account for the three comparisons. A one-way ANOVA to assess differences in PLN histology was performed. A Wilcoxon sign rank test was used to test knee histology parameters.

## Results

### iNOS dependent and independent phases of joint draining lymph node expansion during inflammatory-erosive arthritis

**Fig. 1:**
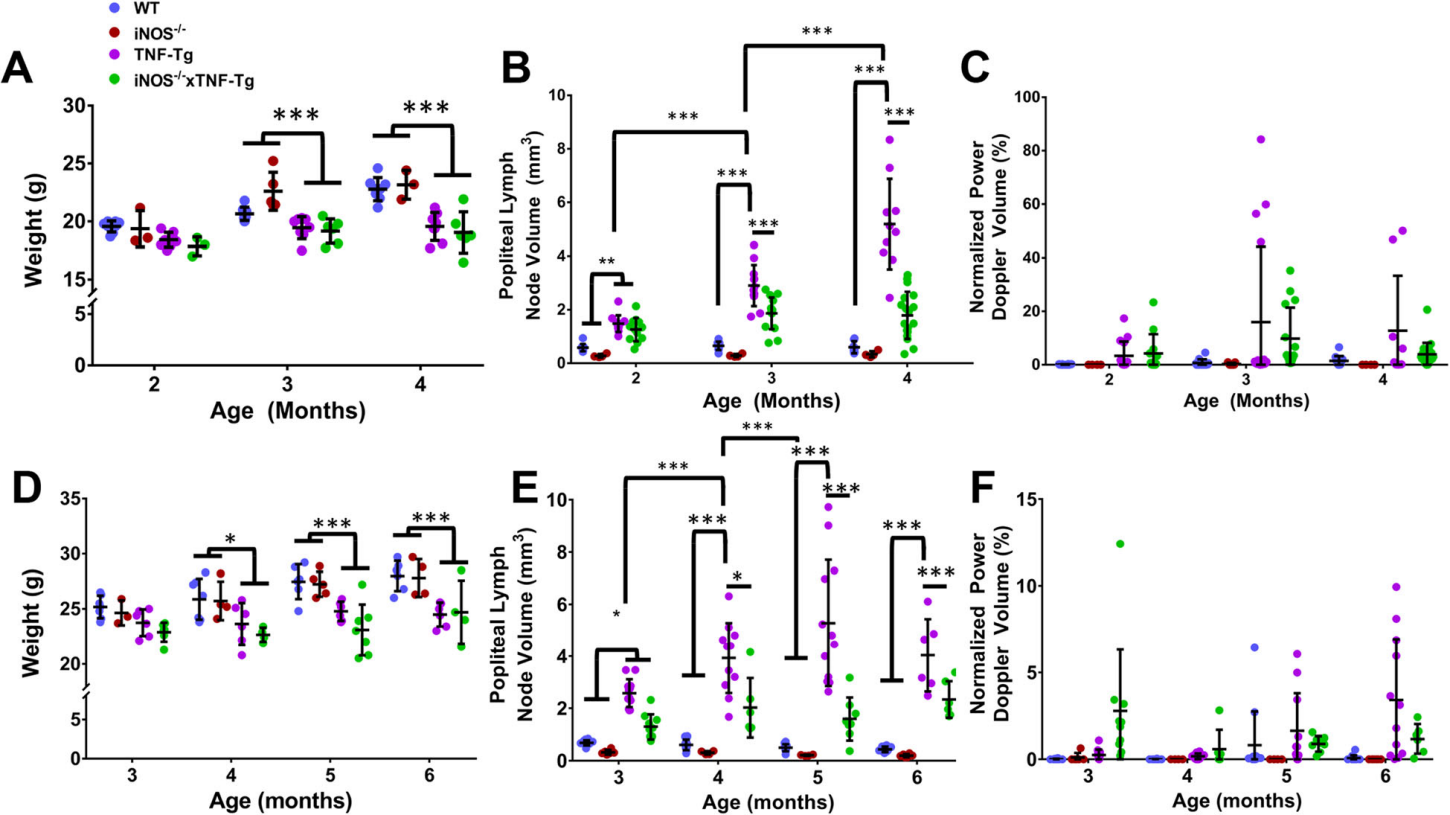
iNOS dependent and independent phases of lymph node expansion in TNF-Tg mice. Female (**a**–**c**) and male (**d**–**f**) mice with the indicated genotype were studied to determine body weight (**a**, **d**), PLN volume via ultrasound (**b**, **e**), and blood flow within the PLN (**c**, **f**) as described in the “Methods” section, and the data are presented for each mouse with mean ± SD for the group. Genetic iNOS ablation had no effect on weight in female mice (**a**). Popliteal lymph node volume was initially increased in both TNF-Tg and iNOS^−/−^× TNF-Tg at 2 months of age in female mice compared to WT and iNOS^−/−^ littermates (**b**). However, at 3 months of age, TNF-Tg PLN volume continues to increase while iNOS^−/−^× TNF-Tg does not, suggesting an iNOS dependent and independent phase of lymph node expansion. Interestingly, there were no significant differences in NPDV over time (**c**). These findings are conserved in male mice such that iNOS^−/−^ does not significantly protect the mice from weight loss, and there are iNOS dependent and independent phases of PLN expansion (**d**–**f**). Statistical analysis: *n* = 4–6 mice per group, *n* = 8–12 PLNs, three-way mixed model, *p < 0.05, **p < 0.01, *p* < 0.001

Previously, we found significant weight loss in TNF-Tg mice with inflammatory-erosive arthritis [11]. To assess the contribution of iNOS to this phenotype, we measured whole body weights over time and found that both female TNF-Tg and iNOS^−/−^× TNF-Tg mice had significantly similar decreased weight at 3 and 4 months of age compared to WT and iNOS^−/−^ (Fig. 1a). We then investigated PLN volume and found both TNF-Tg and iNOS^−/−^× TNF-Tg mice had significantly similar increased PLN volume at 2 months of age. However, at 3 and 4 months of age, TNF-Tg PLN volume continued to increase, while the volume in iNOS^−/−^× TNF-Tg mice was unchanged after 2 months of age (Fig. 1b). Surprisingly, NPDV, a measure of blood flow, was not different between the groups over time (Fig. 1c). In male mice, iNOS^−/−^ did not significantly alter the weight of TNF-Tg mice (Fig. 1d). Additionally, male TNF-Tg and iNOS^−/−^× TNF-Tg mice had increased PLN volume compared to their respective controls at 2 months of age, while TNF-Tg PLN continued to expand in volume over time, but iNOS^−/−^× TNF-Tg PLN did not (Fig. 1e). Interestingly, no differences in NPDV were observed (Fig. 1f). Of note, the individual variation of NPDV in both female and male TNF-Tg mice (Fig. 1c, f) was large, which may influence the statistical analysis of this dataset.

Given the previously described sexual dimorphism in the LNs of the TNF-Tg mouse line [11], we investigated the relationship between female and male iNOS^−/−^ and iNOS^−/−^× TNF-Tg PLN volume. Because male and female TNF-Tg mice demonstrate temporal differences in disease progression, we analyzed the times of peak PLN volumes in TNF-Tg mice (4 months in female mice and 5 months in male mice). Importantly, there were no differences between male and female iNOS^−/−^ or iNOS^−/−^× TNF-Tg mice at peak LN disease (M ± SD: female iNOS^−/−^ 0.33 ± 0.09 vs male iNOS^−/−^ 0.21 ± .02 and female iNOS^−/−^× TNF-Tg 1.79 ± 0.87 vs male iNOS^−/−^× TNF-Tg 1.59 ± 0.82 mm^3^).

**Fig. 2:**
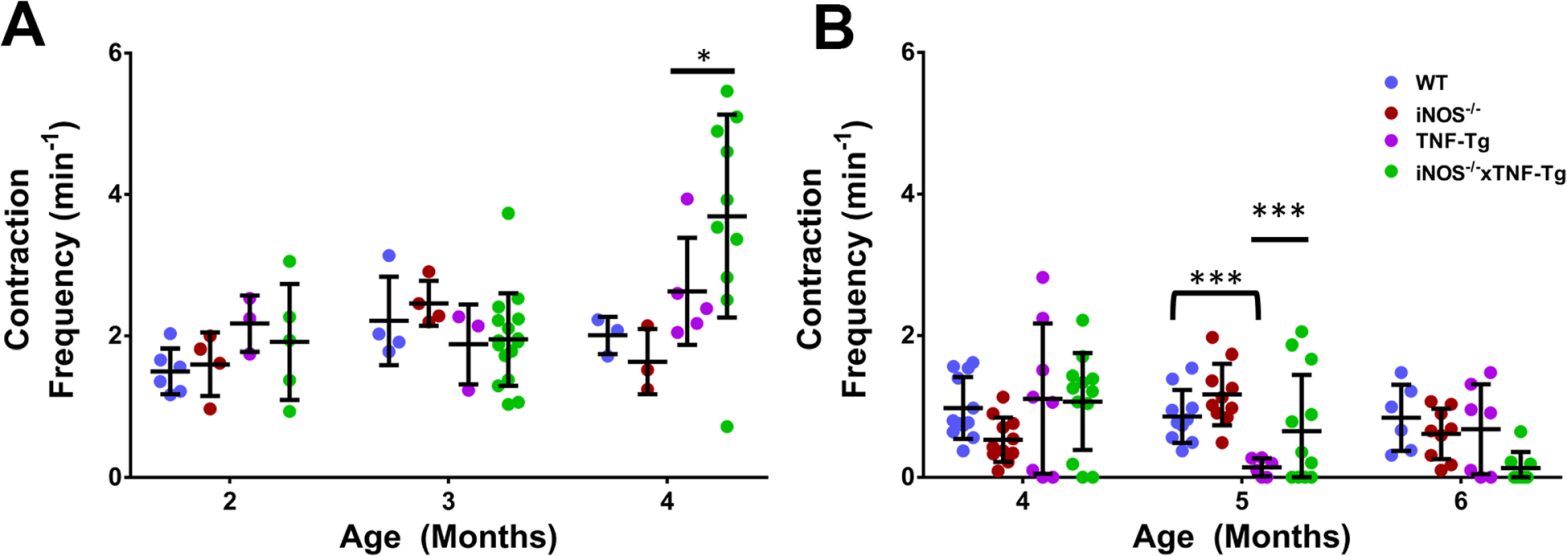
Genetic iNOS ablation preserves lymphatic contractility in TNF-Tg mice. NIR-ICG imaging was performed on female (**a**) and male (**b**) mice with the indicated genotype to determine LV contraction frequency as described in the “Methods” section, and the data are presented for each mouse with mean ± SD for the group. There was no effect on lymphatic contractility in female mice at 2 or 3 months of age; however, at 4 months of age, iNOS^−/−^× TNF-Tg mice had significantly increased lymphatic contractions compared to TNF-Tg mice (**a**). In male mice, TNF-Tg mice had significantly decreased contractions at 5 months of age compared to WT mice, while contractions in iNOS^−/−^× TNF-Tg mice were preserved. Statistical analysis: *n* = 3–10 per group, three-way Mixed Model, *p < 0.05, **p < 0.01, ***p < 0.001

To understand LV function throughout the course of inflammatory-erosive arthritis, and the effect of iNOS^−/−^ on this phenotype, we performed NIR lymphatic imaging in both male and female mice (Fig. 2). NIR imaging revealed an increase in popliteal LV contraction at 4 months of age in female iNOS^−/−^× TNF-Tg mice compared to TNF-Tg mice (Fig. 2a). In male mice, TNF-Tg mice had a significant decrease in contraction frequency at 5 months, while most iNOS^−/−^× TNF-Tg mice maintained their contraction frequency. This relationship disappeared at 6 months, in which iNOS^−/−^× TNF-Tg mice displayed a loss of contraction frequency compared to iNOS^−/−^ mice.

**Fig. 3:**
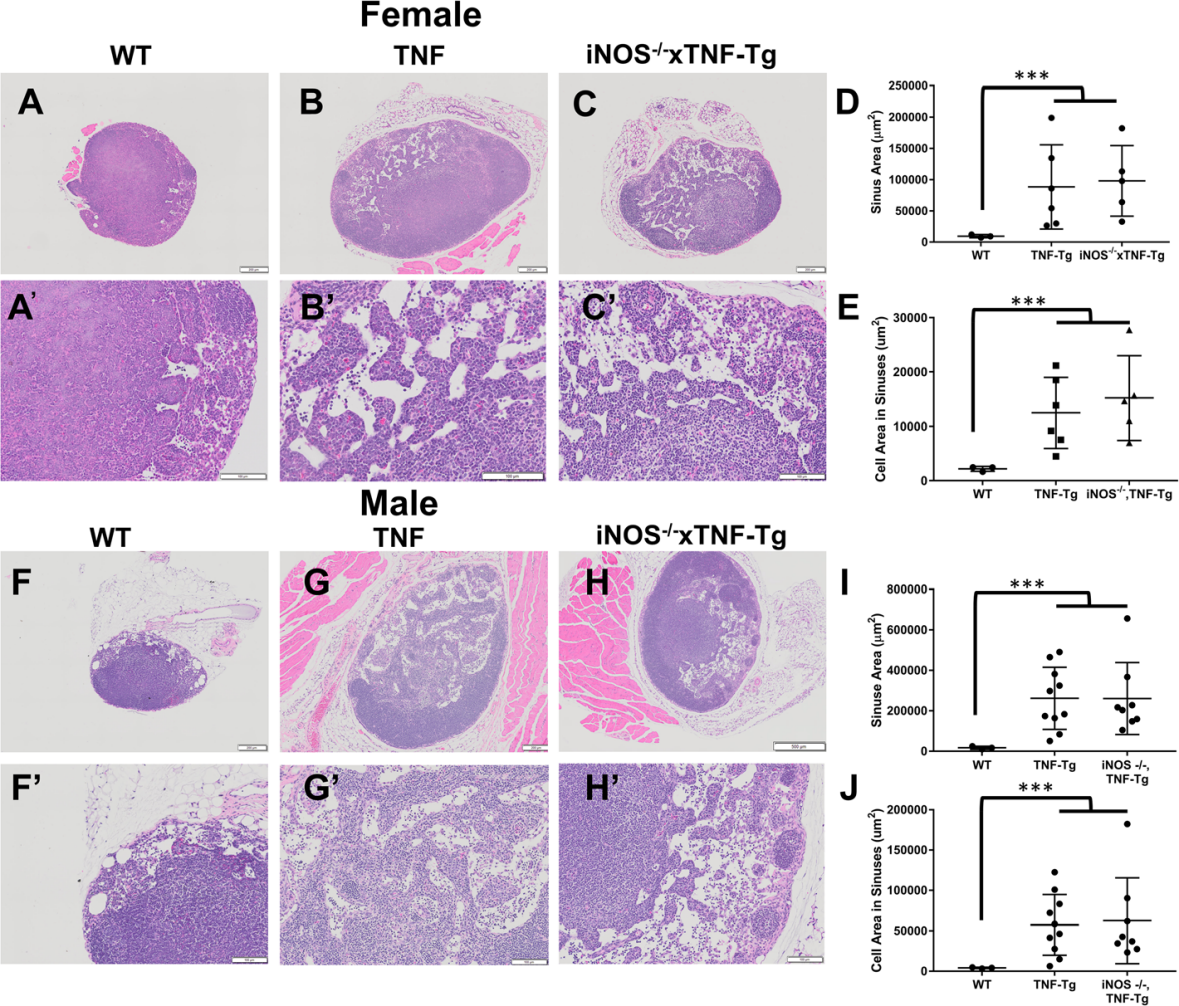
Cellular accumulations in TNF-Tg sinuses are not affected by genetic iNOS ablation. H and E-stained histology of PLN from female (**A**–**E**) and male (**F**–**J**) mice was investigated to assess if iNOS dependent differences in PLN volume are influenced by cells within the PLN sinuses. Representative images at × 4 (**A**–**C**, **F**–**H**) and × 20 (**A′**–**C′**, **F′**–**H′**) of WT (**A**, **A**′, **F**, **F′**), TNF-Tg (**B**, **B′**, **G**, **G′**), and iNOS^−/−^× TNF-Tg (**C**, **C′**, **H**, **H′**) PLN are shown, with histomorphometry (**D**, **E**, **I**, **J**). Note that the results show that both TNF-Tg and iNOS^−/−^× TNF-Tg have increased sinus area and numbers of cells within their sinuses compared to WT mice; however, they are not different from each other. Statistical analysis: *n* = 3–6 per group, one-way ANOVA, ***p < 0.001

**Fig. 4:**
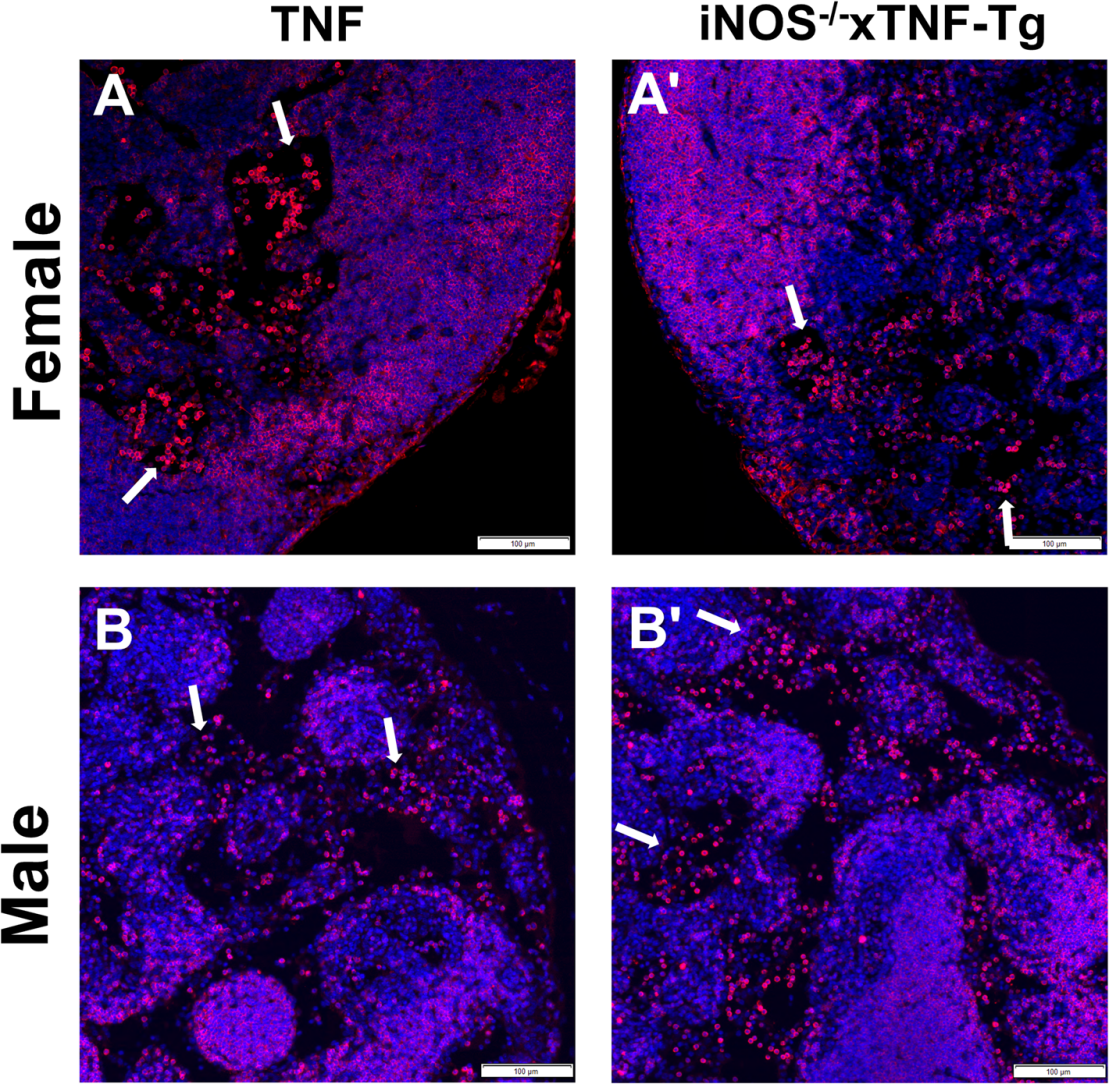
B cell numbers in TNF-Tg sinuses are not affected by genetic iNOS ablation. Immunohistochemistry for B220 (red) with DAPI counter stain (blue) was performed on PLNs from female (**A**) and male (**B**) TNF-Tg (**A**, **B**) and iNOS^−/−^× TNF-Tg (**A′**, **B′**) mice to assess the role of iNOS on B cells within the PLN sinuses. Representative images at × 20 are shown to illustrate similar B cell (arrows) number in TNF-Tg and iNOS^−/−^× TNF-Tg PLN sinuses

In previous work, we described the significant influx of B cells in draining LN and their migration from the follicular area into the LN sinuses [4, 8, 9, 26]. To asses if iNOS plays a role in the LN phenotype described above, we assessed PLN histology from female and male mice (4 and 6 months, respectively). Enlarged LN sinuses were noted in both the TNF-Tg and iNOS^−/−^× TNF-Tg female mice, and significantly, more cells were present in their sinuses compared to WT mice (Fig. 3 A–E). Interestingly, we found no difference in cellular accumulation within the sinuses of PLN from TNF-Tg and iNOS^−/−^× TNF-Tg mice (Fig. 3E). The loss of iNOS in male TNF-Tg mice also had no effect on the cellularity of PLN sinuses (Fig. 3F–J). To assess potential iNOS effects on B cells within the PLN of TNF-Tg mice, we performed immunohistochemistry for B cells and did not observe remarkable differences between TNF-Tg and iNOS^−/−^× TNF-Tg PLNs (Fig. 4A, B). However, as large numbers of B220+ cells were observed in iNOS^−/−^× TNF-Tg PLN sinuses, we conclude that iNOS unlikely regulates the migration of B cell into the sinuses.

### Genetic iNOS ablation protects female mice from TNF-induced synovitis without affecting osteoclast formation

**Fig. 5:**
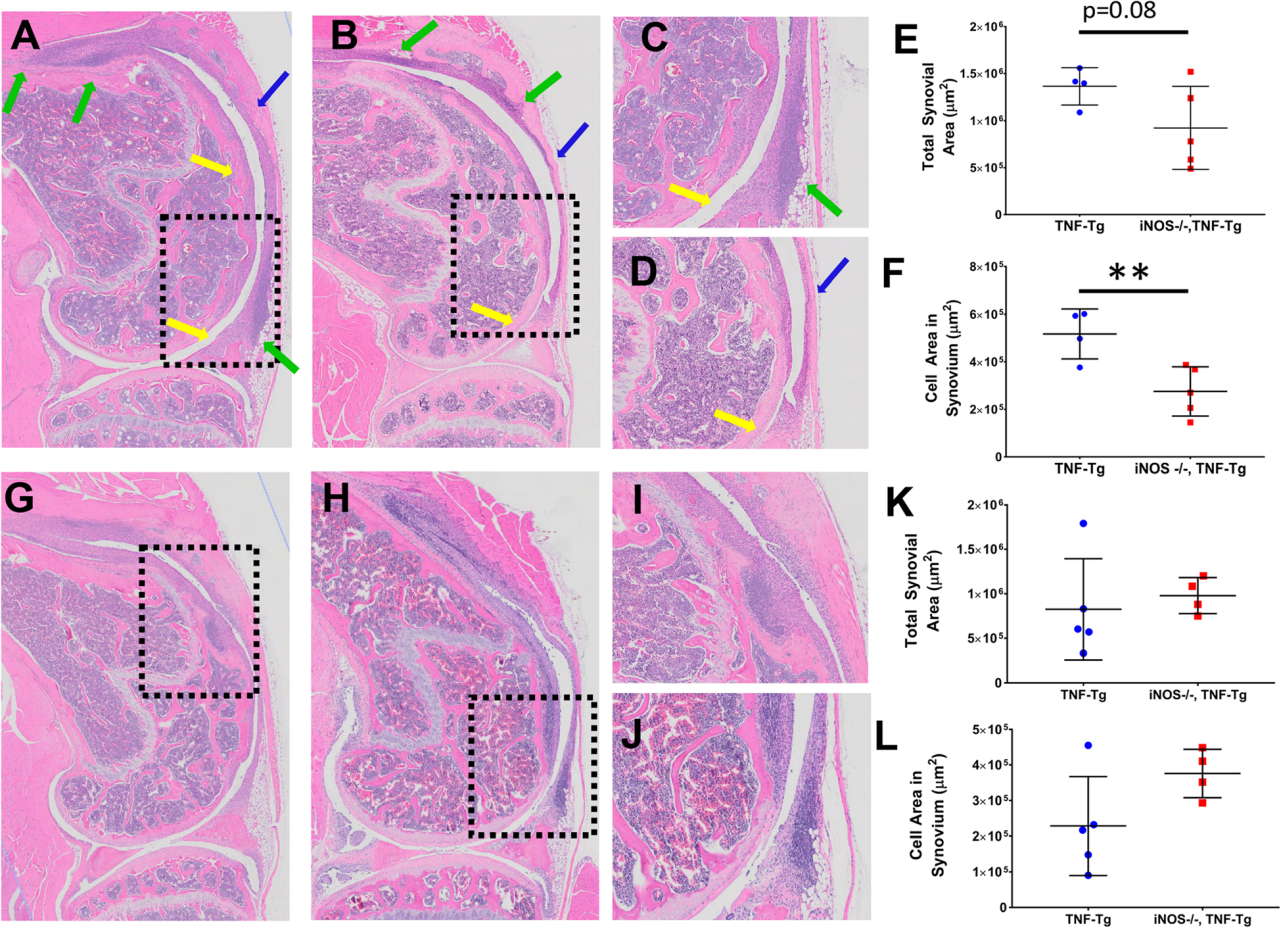
Genetic iNOS ablation protects against cellular accumulations in the synovium of TNF-Tg female, but not in male mice. Representative H and E-stained slides of female TNF-Tg (**a**, **c**) and iNOS^−/−^× TNF-Tg (**b**, **d**), and their corresponding high magnification images (× 50 and × 100). Note the decrease in synovial thickness (blue arrows); the smaller and fewer in number immune cell clusters (green arrows), and decreased articular cartilage invasion (yellow arrows) in the iNOS^−/−^× TNF-Tg knee compared to the TNF-Tg knee. Quantification shows a decreasing trend in total synovial area and cell area within the synovium (**e**, **f**). Interestingly, the male TNF-Tg (**g**, **i**) and iNOS^−/−^× TNF-Tg mice (**h**, **j**) do not display any histomorphometric difference in total synovial area and cellular area in the synovium (**k**, **l**). Statistical analysis: *n* = 4–6 per group, Wilcoxon’s rank-sum test, **p < 0.05

**Fig. 6:**
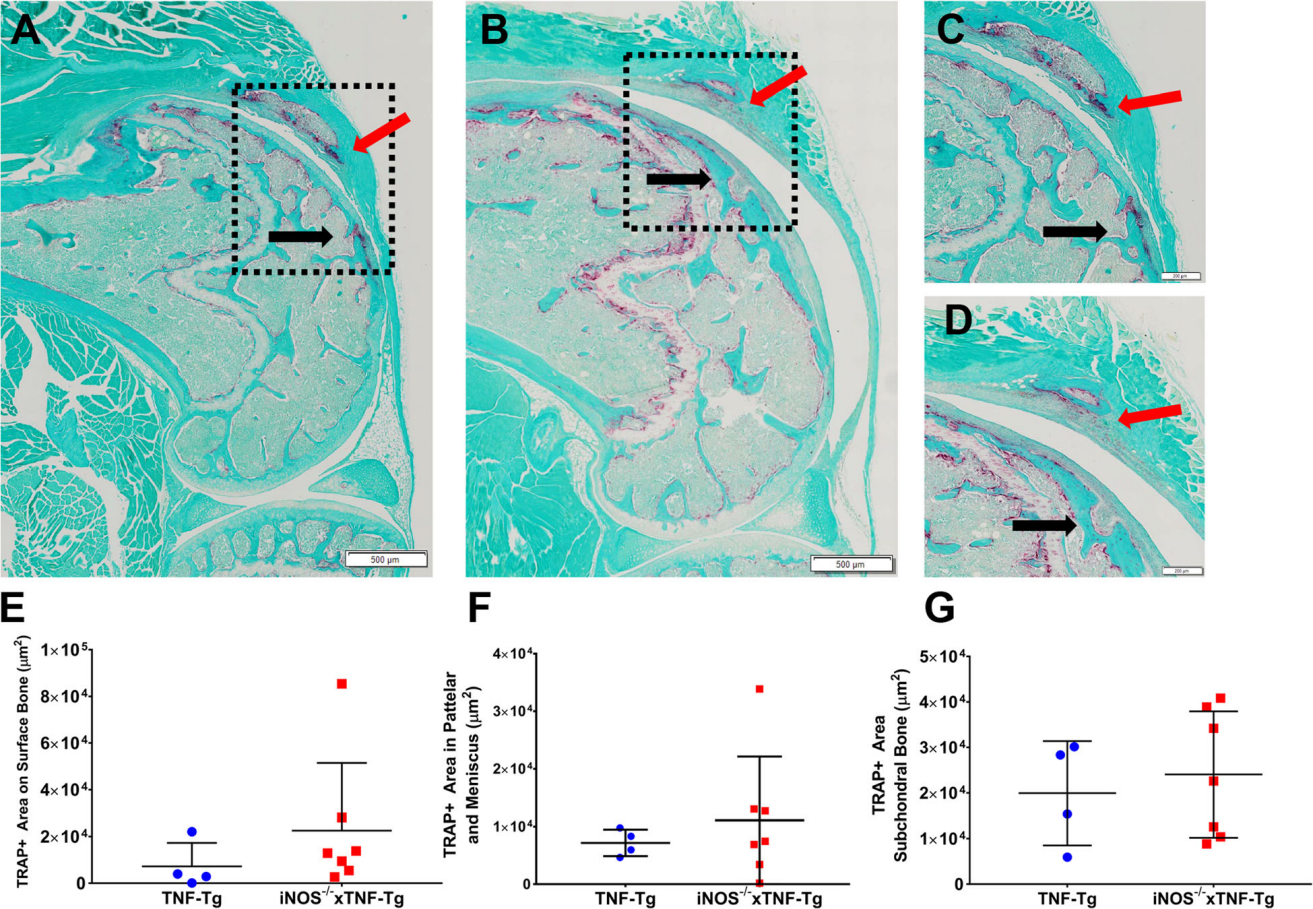
Genetic iNOS ablation has no effect on osteoclast numbers in TNF-Tg female mice. Representative TRAP-stained slides of female TNF-Tg (**a**, **c**) and iNOS^−/−^× TNF-Tg mice (**b**, **d**), and their corresponding high magnification images (× 20 and × 40). Note the TRAP+ area on the surface of the condyle surface (black arrow) and in the patella (red arrows) in both the TNF-Tg and iNOS^−/−^× TNF-Tg mice. No differences in TRAP+ area were detected on the surface, in the patella and meniscus or the subchondral bone area (**e**, **f**, **g**)

To assess the effects of iNOS ablation on TNF-induced inflammatory-erosive arthritis, we performed histomorphometry on the knees from 4-month-old female and 6-month-old male TNF-Tg and iNOS^−/−^× TNF-Tg mice. Histomorphometry of the pannus tissue revealed a trend of decreased synovitis with significantly fewer immune cell clusters in iNOS^−/−^× TNF-Tg compared to TNF-Tg female knees (Fig. 5a–f). However, no iNOS effects on synovitis were observed in male mice (Fig. 5g–l). Osteoclast formation was assessed by the presence of TRAP+ staining, and interestingly, TRAP staining of female knees showed no difference in TRAP+ area in the subchondral space, the surface of the condyle, or the patella and meniscus areas (Fig. 6). Thus, iNOS selectively protects TNF-induced synovitis in female mice, without affecting osteoclast formation.

## Discussion

The mechanistic impact of iNOS in rheumatoid arthritis is very challenging to elucidate due to the pleiotropic and contrasting functions of NO in the maintenance of homeostasis and in other settings, the promotion of inflammation. While initial reports of iNOS inhibition in animal models of acute arthritis were promising [27, 28], clinical trials of selective iNOS inhibitors in early RA patients failed to demonstrate significant efficacy [29]. However, given its potential to alter LV disfunction in RA, we investigated the role of genetic iNOS ablation on draining LNs, LV contraction, and arthritis in TNF-Tg mice with inflammatory-erosive arthritis. Interestingly, here, we identified two novel phases of joint draining LN dynamics, an early iNOS independent phase and a subsequent iNOS dependent phase. These phases were conserved between the sexes (Fig. 1b, e) and were not associated with cellular distributions in PLN at the prospective terminal timepoints (Fig. 3).

Previously, our lab identified distinct phases of PLN volume in TNF-Tg mice that were associated with discrete stages of knee arthritis [7, 11]. In the early phase of disease, PLNs undergo a volumetric enlargement from 3–6× that of WT, which is associated with early arthritis. Subsequently, the LN stochastically collapses via an unknown mechanism, an event associated with end-stage arthritis. The presence of macrophages and iNOS producing lymphatic endothelial cells, and a loss of lymphatic pumping pressure in LV, suggested dysregulation of the lymphatic contractions may influence the LN phenotype, and thus, modulation of iNOS production may alter the disease phenotype [6, 14, 16]. Importantly, we find that genetic iNOS ablation altered this PLN phenotype (Fig. 1) but that lymphatic contractility changes occurred at different timepoints (Fig. 2) suggesting independent mechanisms. These contrasting findings may relate to the known iNOS effects on the expansion phase of the PLN independent of contractions, while vessel contractility and lymph pressure may be controlling the collapsing phase, which our study was not designed to investigate. Consistent with this interpretation is the report of LN expansion in an immunization model which was dependent on C-type lectin 2 (CLEC-2) expressing dendritic cells (DCs) [30]. When CLEC-2 null DCs were adoptively transferred to irradiated, immunized mice, fewer migratory DCs were observed in the draining LN. The lower number of DCs was also associated with reduced rearrangement and enlargement of the fibroblastic reticular network (FRN), as CLEC-2 was found to inhibit podoplanin (PDPN) dependent contractility of the FRN. Further, iNOS ablation reduces DC expansion and may play a role in LN size in the TNF-Tg model [31].

This prevention of LN expansion, however, may not necessarily be beneficial for arthritic disease outcomes in TNF-Tg mice. Our histologic results indicate moderate attenuation of the arthritis phenotype in female mice and no alteration in the phenotype in male mice. The failure to achieve a robust reduction in arthritis may be explained by multiple factors. First, the inflammatory arthritic phenotype in TNF-Tg mice is largely non-antigen driven [4]. Therefore, any alteration in antigen presenting cell egress into the LN may not alter the antigen independent inflammatory reactions occurring in the TNF-Tg mice. Second, NO and iNOS play critical roles in both osteoblast (OB) and osteoclast (OC) activity [32–34]. iNOS is a negative regulator of osteoclast formation and can induce osteoblast proliferation. Inhibition of iNOS may actually increase bone loss in some inflammatory settings. The OC and OB effect would be independent from immune cell egress to the joint draining lymph node via lymphatic transport. Lastly, the iNOS dependent arthritic attenuation may take place at specific timepoints not captured in this study, especially in male mice. The effect in males may be operative at earlier timepoints, and the genetically induced overproduction of TNF may overwhelm any potential benefit at the time of our terminal timepoint.

B cell accumulation in the follicles and sinuses was also shown to be important in expanding and collapsing TNF-Tg LNs [4, 8]. To investigate these cellular accretions, we perform histomorphometry on H and E-stained LNs. Interestingly, we found no difference in cell numbers within the sinuses of TNF-Tg and iNOS^−/−^× TNF-Tg LN, suggesting that at these timepoints, B cell translocation may not play a role in LN expansion. Further, when we qualitatively analyzed B220-stained LNs, there were no remarkable differences between TNF-Tg and iNOS^−/−^× TNF-Tg LNs.

Our study design limited the breadth of our conclusions because we utilized a pan genetic ablation of iNOS and a cell-targeted approach may have elucidated specific cellular effects of iNOS on TNF-induced arthritis. This is particularly important in understanding the role of iNOS on the vascular system as NO is a potent vasodilator, and iNOS was found at increased levels in RA patients and has been implicated in vascular endothelial dysfunction [35]. Further, iNOS has been seen in the aortas of collagen-induced arthritic mice, suggesting a role of iNOS in the vascular system in arthritic mouse models [36]. Lastly, there is a small but statistical difference in WT and iNOS^−/−^ LN volume at baseline when analyzed independent of TNF-Tg. However, there was no difference in TNF-Tg and iNOS^−/−^× TNF-Tg LN volume at the earliest timepoints suggesting that in the setting of inflammation, there is an iNOS independent phase of expansion.

## Conclusion

In conclusion, we identified iNOS independent and dependent phases of LN expansion in an inflammatory-erosive arthritis model. This data suggests iNOS may play a role in LN responses throughout the course of arthritic progression.

## Data Availability

The datasets used and/or analyzed during the current study are available from the corresponding author on reasonable request.

## Abbreviations

CPM: Contractions per minute
FLS: Fibroblast-like synoviocytes
ICG: Indocyanine green
iNOS: Inducible nitric oxide synthase
LNs: Lymph nodes
LV: Lymphatic vessel
NIR: Near-infrared imaging
NO: Nitric oxide
NPDV: Normalized power Doppler volume
PD: Power Doppler
PLN: Popliteal lymph node
RA: Rheumatoid arthritis
ROI: Region of interest
TNF-Tg: Tumor necrosis factor-transgenic
WT: Wild type
